# Dysregulated expression of PSGL-1 and ADAM8 in Sjögren’s disease: potential contribution to disease pathogenesis

**DOI:** 10.3389/fimmu.2026.1848689

**Published:** 2026-07-22

**Authors:** Alejandra Ramos-Manzano, Inés Sánchez-Abad, Esther San Antonio, Miren Uriarte-Ecenarro, Isidoro González-Álvaro, Juan Carlos Saez-Martínez, Marina Dueñas-Ochoa, Javier García-Pérez, Julio Ancochea, Esther F. Vicente-Rabaneda, Santos Castañeda, Ana Urzainqui

**Affiliations:** 1 Immunology Department, Fundación para la Investigación Biomédica (FIB)-Hospital Universitario de La Princesa, Instituto de Investigación Sanitaria (IIS)-Princesa, Madrid, Spain; 2 Medicine Department, Universidad Autónoma of Madrid, Madrid, Spain; 3 Rheumatology Department, Hospital Universitario La Princesa, Instituto de Investigación Sanitaria del Hospital Universitario La Princesa – IIS Princesa, Madrid, Spain; 4 Pulmonology Department, Fundación para la Investigación Biomédica (FIB)-Hospital Universitario de La Princesa, Instituto de Investigacion Sanitaria (IIS)- Princesa, Madrid, Spain

**Keywords:** ADAM8, cellular biomarkers, PSGL-1, systemic sclerosis, Sjögren’s disease, systemic lupus erythematosus

## Abstract

**Background:**

Sjögren’s disease (SjD) is a systemic autoimmune disease showing clinical characteristics similar to systemic lupus erythematosus (SLE) and systemic sclerosis (SSc) at early phases, complicating diagnosis.

**Methods:**

In this cross-sectional study, blood was obtained from 65 SjD, 34 SLE, and 44 SSc patients and 56 healthy donors (HD). Numbers and percentage of circulating leukocytes and expression of HLA-DR, PSGL-1, ADAM8, IL-10 and IFNα were determined by flow cytometry.

**Results:**

Compared to HD, SjD patients showed generalized immune system activation and global reduction of circulating leukocytes, except for plasma cells, which were increased. IFNα levels were reduced in SjD CD16- monocytes, dendritic cells (DC), B cells and basophils, but increased in SjD CD16^+^ monocytes and T cells, showing a positive correlation between IFNα T cell levels and percentage of circulating plasma cells. ADAM8 expression levels were reduced in CD16+ monocytes and B cells. In plasma cells and plasmablasts reduction of ADAM8 expression levels were observed during disease activity. SjD patients had reduced percentage of PSGL-1^+^ neutrophils and increased percentage of PSGL-1^+^ B cells (PS1B), which expressed higher levels of IL-10 than those from HD. Low percentage of PSGL-1-expressing neutrophils, basophils, T cells and NK cells and low PSGL-1 expression level in pDC associated with the presence of cutaneous manifestations. Low PSGL-1 expression level in basophils, cDC and both CD16^-^ and CD16^+^ monocytes was associated with the presence of anti-SSB autoantibodies. Remarkably, we found two cellular biomarkers: PS1B/%pDC ratio (PPr) distinguished SjD patients from HD (accuracy: 78%) and from SSc (accuracy: 84%) and PS1B discriminated SjD from SLE (accuracy: 70%). Both biomarkers improved diagnostic accuracy when combined with current serological markers.

**Conclusions:**

Dysregulated PSGL-1 and ADAM8 expression in SjD circulating cells is associated with important clinical manifestations in SjD patients, supporting their potential relevance as therapeutic targets in this disease. Additionally, we found PS1B and PPr as new cellular biomarkers that could help improve SjD diagnostic accuracy, particularly in seronegative or ambiguous cases during the early phase of disease.

## 1 Introduction

Sjögren’s disease (SjD) is a chronic autoimmune disorder characterized by chronic inflammation, lymphocytic infiltration and progressive destruction of exocrine glands. Extraglandular involvement can lead to severe complications, including an increased risk of non-Hodgkin lymphoma (1). Clinical heterogeneity and overlap with other connective tissue diseases (CTDs) such as systemic lupus erythematosus (SLE) and systemic sclerosis (SSc) further complicate early recognition (2).

Current SjD diagnosis combines clinical features with serology and histopathology disturbances. The 2016 American College of Rheumatology (ACR)-European League Against Rheumatism (EULAR) criteria use a weighted score of five items, with anti-SSA/Ro seropositivity and focal lymphocytic sialadenitis on minor salivary gland biopsy assigned the highest weight (3). Since anti-SSA/Ro antibodies are absent in up to one-third of patients and may also appear in other autoimmune diseases (4), biopsy remains the most specific test (5). Therefore, the search for minimally invasive biomarkers has considerably increased. Omics platforms such as RNA sequencing or salivary proteomics have identified SjD candidate biomarkers (6, 7), but they require specialized infrastructure and are expensive, while blood-based approaches are cheaper and more accessible.

IFNα and IL-10 have been described to participate in SjD pathogenesis. In fact, patients show an overexpression of IFN-inducible genes in blood and salivary glands (8). However, most evidence in SjD is transcriptional with limited protein-level validation. Although plasmacytoid dendritic cells (pDC) have been considered the major IFNα producers (5), other populations, including cDC, monocytes or B cells have been reported to produce type I IFN when expose to different pathogens (9). Interestingly, levels of IL-10 in saliva and serum of patients with SjD are high and correlate with disease activity (10, 11). IFNα can prime immune cells to confer pro-inflammatory properties to IL-10 (12), suggesting that IFNα/IL-10 imbalance may sustain autoimmunity through unknown mechanisms.

Regarding adhesion molecules, they are underexplored in SjD. P-selectin glycoprotein ligand-1 (PSGL-1) is a glycoprotein expressed in all leukocytes that mediates leukocyte trafficking to the extravascular space, but also functions as an immune checkpoint, shaping immune tolerance (13, 14). In addition, A disintegrin metalloproteinase 8 (ADAM8) regulates PSGL-1 function through proteolytic cleavage of PSGL-1 (15). Alterations of PSGL-1 and ADAM8 expressions have been reported in several CTDs. The expression of PSGL-1 on neutrophils and monocytes of active SLE patients is decreased (16, 17), and in SSc patients, monocytes, dendritic cells and T cells show increased PSGL-1 expression (18). Animal studies further support its relevance, as the absence of PSGL-1 in mice induces SSc-like features (19). In our previous works, we found alterations in the serum levels of PSGL-1 and ADAM8 in SLE and SSc patients, enabling the identification of novel serum biomarkers that could distinguish healthy donors (HD) from SLE and SSc patients and between these two diseases (20). These findings highlight the PSGL-1/ADAM8 axis as a shared altered pathway in some CTDs, raising the question of whether similar alterations may also occur in SjD.

Therefore, in this study, we explored whether PSGL-1 or ADAM8 expression is altered in SjD patients, which might contribute to the disruption of immune system homeostasis and, consequently, to SjD pathogenesis. Using multiparameter flow cytometry, we characterized blood immune populations in SjD patients focusing on PSGL-1 and ADAM8 expression as well as HLA-DR, IFNα and IL-10 expression and further explored their associations with clinical parameters. Given the necessity for more reliable biomarkers, we evaluated potential biomarkers to aid in the diagnosis of SjD and its differentiation from other CTDs.

## 2 Materials and methods

### 2.1 Patients and samples

This cross-sectional study consecutively recruited a total of 65 SjD, 34 SLE and 44 SSc patients, in routine clinical care at the Rheumatology Unit of Hospital Universitario de La Princesa (HUP, Madrid, Spain), and 56 age- and sex-matched HD. This study was performed following the “STrengthening the Reporting of OBservational studies in Epidemiology” (STROBE) recommendations. The study protocol was approved by the HUP Ethics Committee (Reference numbers: N° 4916: Acta 22/24, N° 3106: Acta 11/17, N° PI-758: Acta 13/14, N° PI-548: Acta 7/12), following the Helsinki declaration. All patients had established diseases and provided written informed consent in compliance with Spanish data-protection legislation.

For each subject, peripheral blood was collected. Then, an aliquot was analyzed with an automated cell blood counter, and the remaining blood was used for flow cytometry studies. All samples were processed immediately after extraction. To minimize systematic batch effects, HD and patient samples were alternated and analyzed by the same operator. SjD patients fulfilled the 2016 ACR–EULAR classification criteria (3). Patients with “associated SjD” (SjD associated with other CTDs), unclear diagnoses or active infectious diseases were excluded. Sample size was determined by available patients in the Outpatient Clinic which signed the informed consent during the recruitment period. SjD activity was assessed with the EULAR Sjögren’s syndrome disease activity index (ESSDAI) score, with scores <5 or ≥5 indicating low and moderate-high activity, respectively (21). SLE and SSc patients were recruited between 2013 and 2016. SLE diagnosis was determined by EULAR/ACR classification criteria (22) and disease activity was assessed with SLE Disease Activity Index (SLEDAI) (23). SSc patients were classified following the EULAR/ACR criteria (24) into limited cutaneous (lcSSc) or diffuse cutaneous (dcSSc) subtypes. Clinical and treatment data are summarized in **Supplementary Tables 1–3**.

### 2.2 Flow cytometry analysis

Whole-blood cells staining was performed on fresh blood without prior freezing. 100µL of peripheral blood was incubated with 10µg/mL human γ-globulin (Sigma-Aldrich) for 10 min at 4 °C to block Fc receptors. Then, cells were incubated for 20 min at 4°C in the dark with directly conjugated antibodies anti-HLA-DR, anti-PSGL-1 and purified anti-human ADAM8, together with antibodies used to identify different leukocyte populations. For ADAM8 detection, samples were incubated for 20 min at 4 °C with a conjugated secondary antibody after surface staining. Specific monoclonal antibodies directed to human CD3, CD11c, CD14, CD16, CD19, CD38, CD56 and CD123 were used for identifying leukocyte populations. Red blood cells were lysed and leukocytes fixed and permeabilized using BD FACS Lysing Solution (BD Biosciences) for 15 min at room temperature in the dark. Antibody details and dilutions are listed in **Supplementary Table 4**.

For intracellular cytokine detection (IL-10 and IFNα), unstimulated blood cells, following surface staining and permeabilization, were incubated with human γ-globulin as described above. Then, cells were incubated with directly labelled anti-IL-10 or anti-IFNα antibodies for 20 min at 4 °C. Finally, CountBright™ Absolute Counting Beads (Invitrogen, Waltham, MA, USA) were added per manufacturer instructions to calculate absolute cell counts per mL of blood.

To determine positive expression of target molecules, blood cells were labelled with isotype controls (**Supplementary Figures 1A, B**). Additionally, Fluorescence-Minus-One (FMO) or intracellular isotype controls were included for determining the positivity of intracellular molecules (**Supplementary Figure 1C, D**).

Data were acquired on a BD FACSCanto II (BD Biosciences). Instrument performance and day-to-day variability were monitored by daily quality control using Cytometer Setup & Tracking (CS&T) beads. Before each acquisition, BD Rainbow Calibration Particles (BD Biosciences) were used to verify PMT voltages and inter-run stability. Both percentage of expressing cells and expression levels, assessed as mean fluorescence intensity (MFI) were analyzed in each population using FACSDiva Software (BD Biosciences).

### 2.3 Gating strategy

All leukocyte populations were defined in the gate of singlets. Neutrophils were identified as CD16^+^. Monocytes were identified as CD14^+^ and, classical (CD16^-^ CD14^+^ or CD16^-^ monocytes) and non-classical monocytes as (CD16^+^ CD14^+^ or CD16^+^ monocytes) were analyzed. Basophils were identified in the low SSC CD14^-^ gate, as HLA-DR^-^CD123^+^. pDC and conventional dendritic cells (cDC) were identified after excluding basophils: pDC as HLA-DR^+^, CD11c^-^, CD123^high^ and cDC as HLA-DR^+^, CD11c^+^, CD123^low^. Gating strategy for myeloid populations is shown in **Supplementary Figure 2A**.

Within the lymphoid gate (low SSC and FSC), B cells and T cells were defined as CD19^+^ and CD3^+^, respectively. Natural killer (NK) cells were identified as CD56^+^ within the CD19^-^CD3^-^ double-negative fraction. Plasmablasts and plasma cells were defined within the CD19^+^ gate as CD19^+^CD38^+^ and CD19^low^CD38^+^, respectively. Gating strategy for lymphoid populations is shown in **Supplementary Figure 2B**.

### 2.4 Automated blood cell counter

As a complementary method to flow cytometry, 50µL of whole blood were analyzed in a Nihon Kohden NK-6450 hematology analyzer (RAL, Barcelona, Spain) to obtain total and differential cell counts of each sample.

### 2.5 ELISA assays

Serum concentrations of PSGL-1 and ADAM8 were quantified using commercially available ELISA kits. PSGL-1 levels were measured using the Human PSGL-1 (P-Selectin Glycoprotein Ligand 1) ELISA Kit (ELK Biotechnology, TX, USA; Cat. No. LK5261), whereas ADAM8 levels were determined using the Human ADAM8 (Disintegrin and metalloproteinase domain-containing protein 8) ELISA Kit (FineTest, Wuhan, China; Cat. No. EH0981). Prior to analysis, serum samples were thawed at room temperature and homogenized thoroughly to ensure uniformity. All assays were performed according to the manufacturers’ instructions, and absorbance was measured using a GloMax Discovery microplate reader (Promega, Madison, WI, USA).

### 2.6 Statistical analysis

#### 2.6.1 Data preprocessing and group comparisons

Clinical variables were dichotomized (e.g., no treatment/treatment). Flow cytometry data were analyzed as continuous variables. Data normality were determined using the Shapiro–Wilk or Kolmogorov–Smirnov tests and homoscedasticity was evaluated using Levene’s test. For comparisons between two independent groups, either Student’s t-test or the Mann–Whitney U test was applied, depending on the data distribution. For bivariate correlation analysis, Spearman’s rank correlation coefficient (ρ) was calculated. Statistical significance was defined at p-value < 0.05.

#### 2.6.2 Diagnostic performance evaluation

Receiver operating characteristic (ROC) curve analyses were performed to evaluate the discriminatory performance of the different models. The area under the curve (AUC) was calculated as a measure of diagnostic accuracy. Comparisons between AUCs from correlated ROC curves were performed using the DeLong test, using the pROC package v1.18.5 in RStudio.

To evaluate the discriminative ability of our quantitative variables in distinguishing patient groups, we performed binary logistic regression (BLR) analyses. Cases with missing values were excluded from the respective analyses. First, variable selection was conducted using backward stepwise selection based on the likelihood ratio. The selected variables were included individually in BLR models and adjusted for the potential confounders age and sex. The optimal cut-off for each model was determined using Youden’s index (YI) derived from ROC curve analysis, by selecting the point with the highest YI. As internal validation, we performed 5-fold cross-validation and the mean ± SEM of sensitivity, specificity, accuracy, and AUC was reported. Subsequently, model performance was evaluated with 4-fold cross-validation with five repetitions yielding a total of 20 resampling iterations to estimate predictive performance. This approach was applied to the candidate biomarkers and classical disease-specific autoantibodies, depending on the BLR tested, to compare their diagnostic performance. All statistical analyses were performed in RStudio using the caret package v7.0-1 (25). For multiple comparisons, we applied one-way ANOVA with Dunnett’s *post hoc* test or the Kruskal-Wallis test followed by Dunn’s *post hoc* test, as appropriate.

Figures were generated using Prism v8.0.2 (GraphPad Software, La Jolla, CA, USA) or RStudio.

## 3 Results

### 3.1 SjD patients show reduced circulating immune cell counts with selective alterations in immune cell distributions

**Figure 1:**
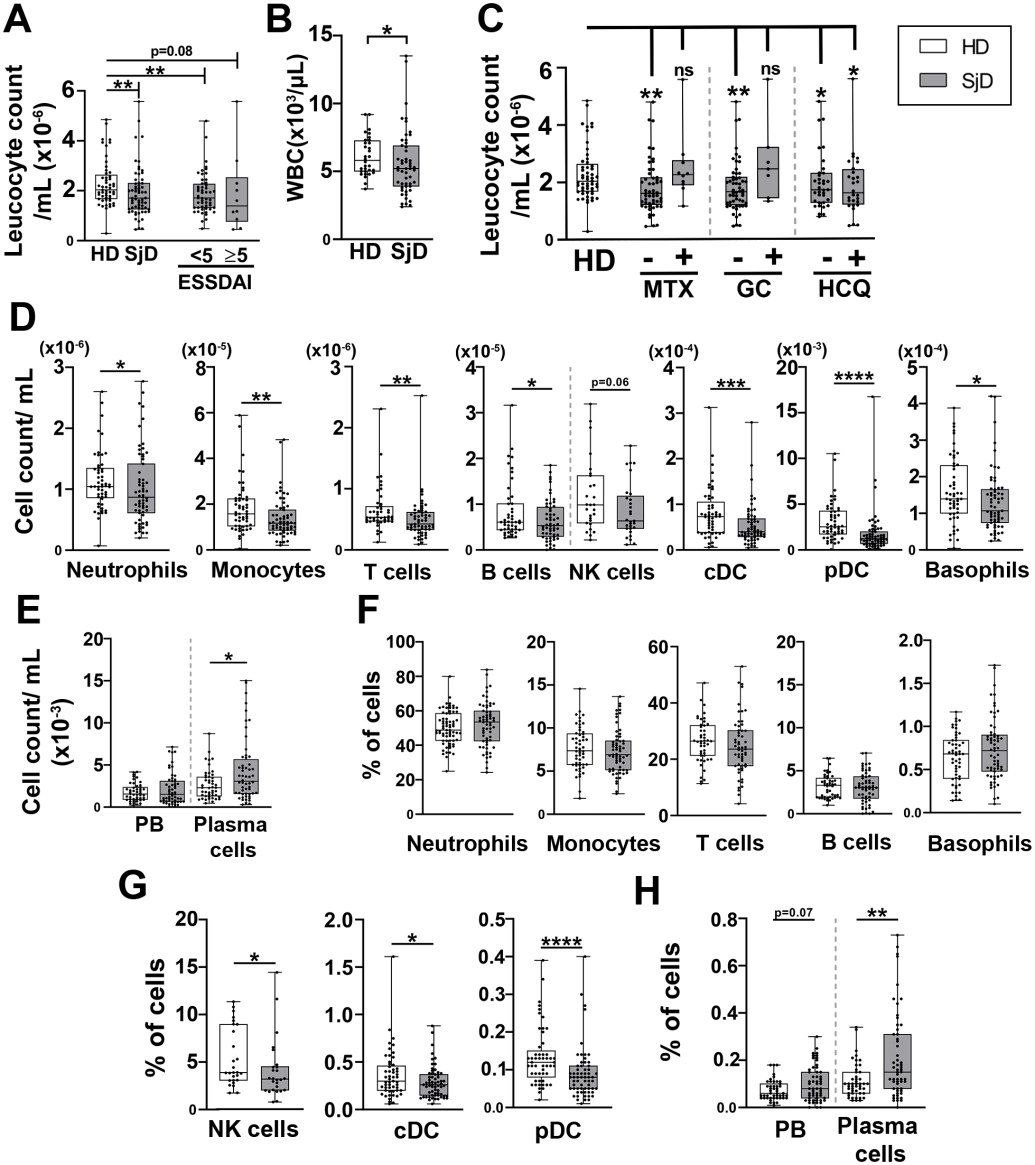
Distribution of circulating immune cells in blood of HD and SjD patients. **(A, B)** Number of circulating immune cells/mL analyzed by flow cytometry, including ESSDAI stratification **(A)**, or by automated hematology analyzer **(B)**. **(C–H)** Analysis by flow cytometry of the number and percentage of circulating cells: **(C)** Leukocyte count/mL, in patients without treatment (–) or with treatment (+). **(D)** Cell count/mL of circulating immune cell populations. **(E)** Cell count/mL of plasmablasts and plasma cells. **(F)** Percentage of circulating immune cell populations. **(G)** Percentage of circulating NK cells, cDC and pDC. **(H)** Percentage of circulating plasmablasts and plasma cells. Percentage and cell counts are relative to singlet cell number. *p<0.05, **p<0.01, ***p<0.001, ****p<0.0001. cDC, Conventional dendritic cells; ESSDAI, EULAR Sjögren’s Syndrome Disease Activity Index; GC, Glucocorticoids; HCQ, Hydroxychloroquine; HD, Healthy donors; MTX, Methotrexate; PB, plasmablasts; pDC, Plasmacytoid dendritic cells; SjD, Sjögren’s disease; WBC, White Blood Cells. Sample size: **(A)** HD n=53, SjD n=63 (ESSDAI<5 n=52, ESSDAI≥5 n=10). **(B)** HD n=32, SjD n=52. **(C)** (–): n=35-60; (+): HCQ n=28, MTX n=10, GC n=7. **(D–H)** NK cells: HD n=27, SjD n=26-29. Myeloid populations: HD n=52-53, SjD n=63. B and T cells, plasmablasts and plasma cells: HD n=48, SjD n=57.

To characterize systemic immune alterations in patients with SjD, we first analyzed the total number of circulating cells per milliliter of blood. Total circulating cell counts were reduced in SjD patients compared to HD, as measured by both flow cytometry (**Figure 1A**) and an automated blood cell counter (**Figure 1B**). We also observed lower hematocrit percentage (**Supplementary Figure 3A**) and a trend toward lower hemoglobin (p=0.06) (**Supplementary Figure 3B**), in SjD patients than in HD. However, the counts of red blood cells and platelets were similar in HD and SjD patients (**Supplementary Figures 3C, D**). Importantly, we found that leucocyte reduction was similar in our cohort of patients with ESSDAI <5 and those with ESSDAI ≥5 (**Figure 1A**). The same trend was found with drug treatments (**Figure 1C**), as patients not receiving immunomodulatory therapy also showed reduced leukocyte counts. A deeper analysis showed that neutrophils, monocytes, T cells, B cells, dendritic cells and basophils were reduced in SjD patients compared to HD (**Figure 1D**) and a trend toward a decrease of NK cell counts (p=0.06). In contrast, the plasmablasts cell count remained unchanged, whereas plasma cells were elevated in SjD patients (**Figure 1E**).

Examination of population distributions as percentages showed that neutrophils, monocytes, T cells, B cells and basophils were similar between SjD and HD (**Figure 1F**). However, SjD patients exhibited reduced percentages of NK cells and dendritic cells, both cDC and pDC (**Figure 1G**). Conversely, the percentage of plasmablasts tended to increase (p=0.07), and the percentage of plasma cells was elevated in SjD patients compared to HD (**Figure 1H**).

### 3.2 Analysis of HLA-DR and IFNα expression in circulating immune cells of SjD

**Figure 2:**
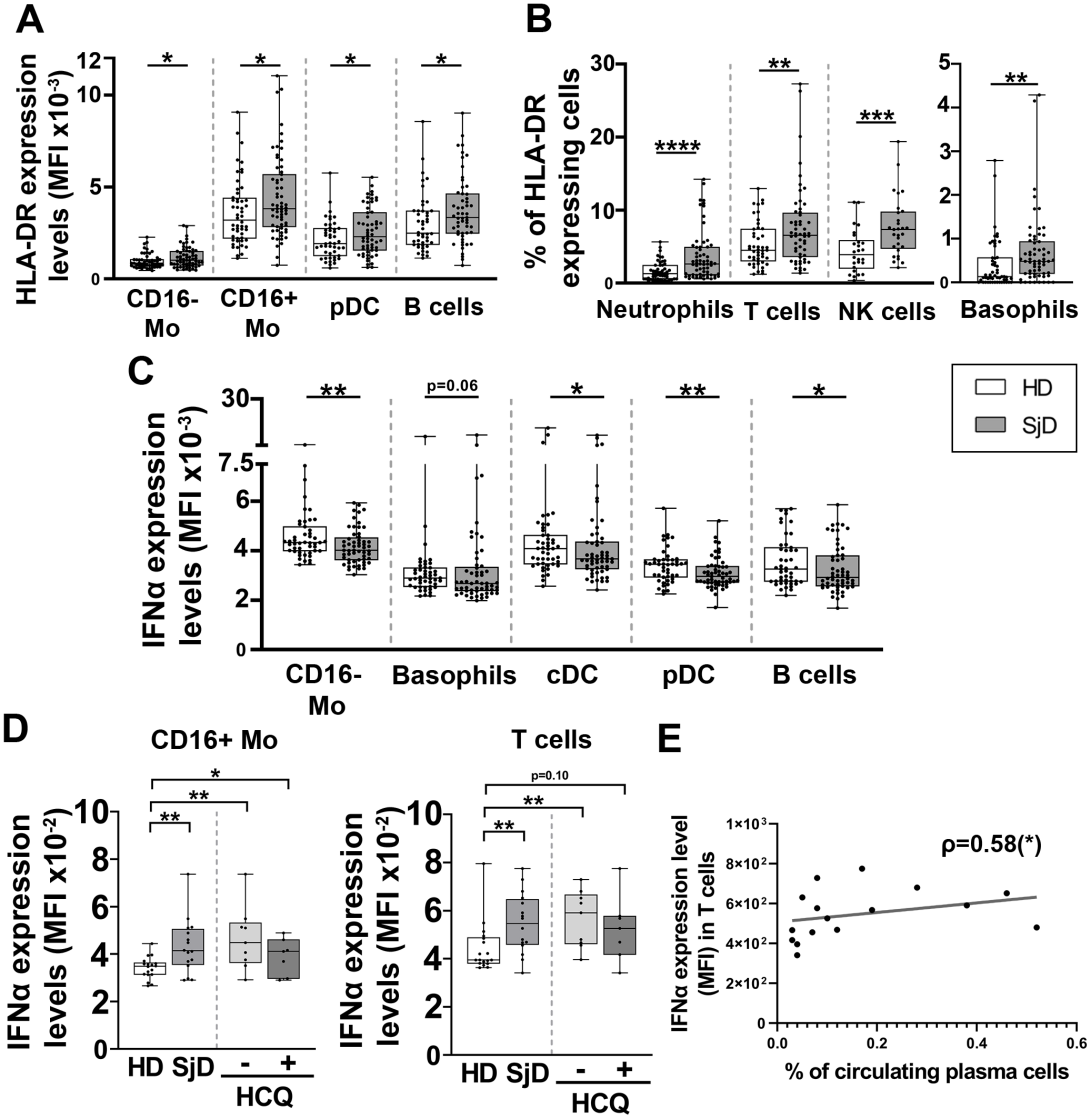
HLA-DR and IFNα expression in SjD patients and HD. **(A)** HLA-DR MFI in different circulating immune populations. **(B)** Percentage of HLA-DR^+^ cells in different circulating immune populations. **(C)** MFI of IFNα expression in circulating immune cell populations. **(D)** MFI of IFNα in CD16^+^ monocytes and T cells, including stratification according to HCQ treatment. **(E)** Correlation between IFNα expression levels in T cells and the % of circulating plasma cells in SjD patients. Percentages of plasma cells are relative to singlet cell number. Bivariate correlation was assessed using Spearman’s rank correlation coefficient. *p<0.05, **p<0.01, ***p<0.001, ****p<0.0001. cDC, Conventional dendritic cells; CD16^-^ Monocytes, Classical monocytes; CD16^+^ Monocytes, Non-classical monocytes; HD, Healthy donors; HCQ, Hydroxychloroquine; pDC, Plasmacytoid dendritic cells; SjD, Sjögren’s disease. Sample sizes: **(A–C)** NK cells: HD n=27, SjD n=26-29. Myeloid populations: HD n=52-53, SjD n=63. B and T cells: HD n=48, SjD n=57. **(D)** CD16^+^ Monocytes and T cells: HD n=18, SjD n=16; HCQ (–) n=9, HCQ (+) n=7.

To study immune system cells activation in SjD patients, we evaluated membrane expression of HLA-DR. HLA-DR expression levels were increased among professional antigen-presenting cells, including CD16^-^ and CD16^+^ monocytes, pDC and B cells (**Figure 2A**). The percentage of HLA-DR^+^ cells was elevated among non-professional antigen-presenting cells such as neutrophils, T cells, NK cells and basophils of SjD patients (**Figure 2B**).

Based on the established role of type I interferons, particularly IFNα, in SjD pathogenesis, we analyzed IFNα expression level (MFI) across multiple circulating immune cell subsets. We found that, in SjD patients, IFNα MFI was reduced in CD16^-^ monocytes, cDC, pDC and B cells, with a trend toward reduced expression in basophils (p=0.06) (**Figure 2C**) and no changes in neutrophils and NK cells (**Supplementary Figure 2E**), whereas T cells and CD16^+^ monocytes were the only populations showing an increased IFNα MFI compared to HD (**Figure 2D**). Stratification by HCQ treatment showed that the difference compared to HD persisted in CD16^+^ monocytes of patients treated with HCQ, whereas it was lost in T cells (p=0.10). However, the differences between patients receiving HCQ and those not receiving HCQ were not statistically significant.

Type I interferons have been reported to influence various aspects of B cell function, including migration and differentiation to plasma cells (26). Therefore, we next assessed whether IFNα expression in T cells was associated with changes in the B cell compartment observed in SjD patients. We found that IFNα MFI in T cells positively correlated with the percentage of circulating plasma cells in SjD patients (ρ=0.58) (**Figure 2E**), a population that is increased in these patients (**Figure 1H**).

### 3.3 Altered ADAM8 expression in circulating leukocytes of SjD patients and its association with ESSDAI and MTX treatment

**Figure 3:**
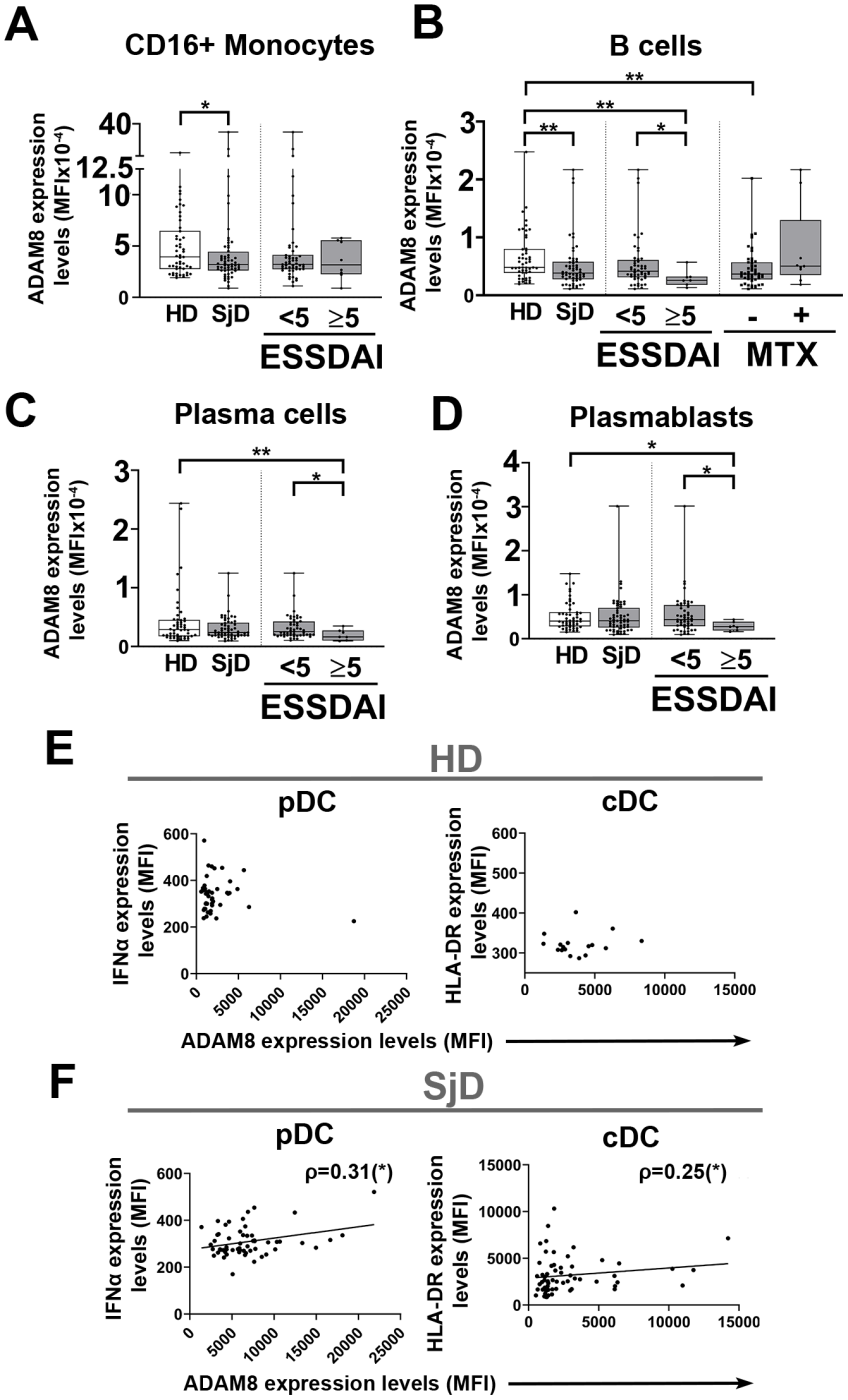
ADAM8 expression levels in circulating leukocytes of SjD patients. **(A)** ADAM8 expression levels (MFI) of CD16^+^ monocytes and its association with ESSDAI. **(B)** ADAM8 expression levels (MFI) of B cells and its association with ESSDAI and MTX. **(C)** ADAM8 expression levels (MFI) of plasma cells and its association with ESSDAI. **(D)** ADAM8 expression levels (MFI) of plasmablasts and its association with ESSDAI. **(E, F)** Correlation between ADAM8 and IFNα expression levels in pDC (left panel) and between ADAM8 and HLA-DR expression levels in cDC (right panel) in **(E)** HD and **(F)** SjD patients. Bivariate correlations were assessed using Spearman’s rank correlation coefficient. *p<0.05, **p<0.01. CD16^+^ Monocytes, Non classical monocytes; HD, Healthy donors; SjD, Sjögren’s disease; MTX, Methotrexate; ESSDAI, EULAR Sjögren’s Syndrome Disease Activity Index. Sample sizes: **(A–D)** HD n=51, (ESSDAI<5): n=51, (ESSDAI≥5): n=7. **(B)** MTX (–) n=50, MTX (+) n=9. **E)** pDC: n=47, cDC: n=50 **F)** pDC: n=57, cDC: n=60.

The expression of ADAM8, metalloprotease that associates with and cuts PSGL-1, in circulating immune cells from SjD patients was also examined. ADAM8 expression was reduced in CD16^+^ monocytes of SjD patients (**Figure 3A**), without statistically significant difference between patients with ESSDAI <5 score and ESSDAI ≥5 score. In addition, B cells from SjD patients also showed reduced ADAM8 expression than those from HD (**Figure 3B**) and was lower during disease activity: patients with ESSDAI≥5 had lower ADAM8 MFI than those with ESSDAI<5 (**Figure 3B**). Notably, we found that treatment with MTX normalized ADAM8 expression in B cells to levels comparable to those of HD. Additionally, although ADAM8 expression levels in neutrophils, CD16^-^ monocytes, basophils, cDC, pDC, T cells and NK cells, were similar in SjD patients and HD (**Supplementary Figure 3F**), a deeper analysis revealed that plasma cells and plasmablasts exhibited reduced ADAM8 expression levels during disease activity (**Figures 3C, D**). Furthermore, serum ADAM8 levels were similar in SjD patients and HD (p=0.53) (**Supplementary Figure 4A**). In addition, no differences were observed between patients with ESSDAI <5 or ≥5.

Interestingly, we found that although there is no correlation between ADAM8 expression level and IFNα or HLA-DR levels in HD (**Figure 3E**), in SjD patients there is a positive correlation between ADAM8 and IFNα expression levels in pDC as well as between ADAM8 and HLA-DR expression in cDC (**Figure 3F**).

### 3.4 PSGL-1 expression in circulating cells from SjD patients and clinical associations

**Figure 4:**
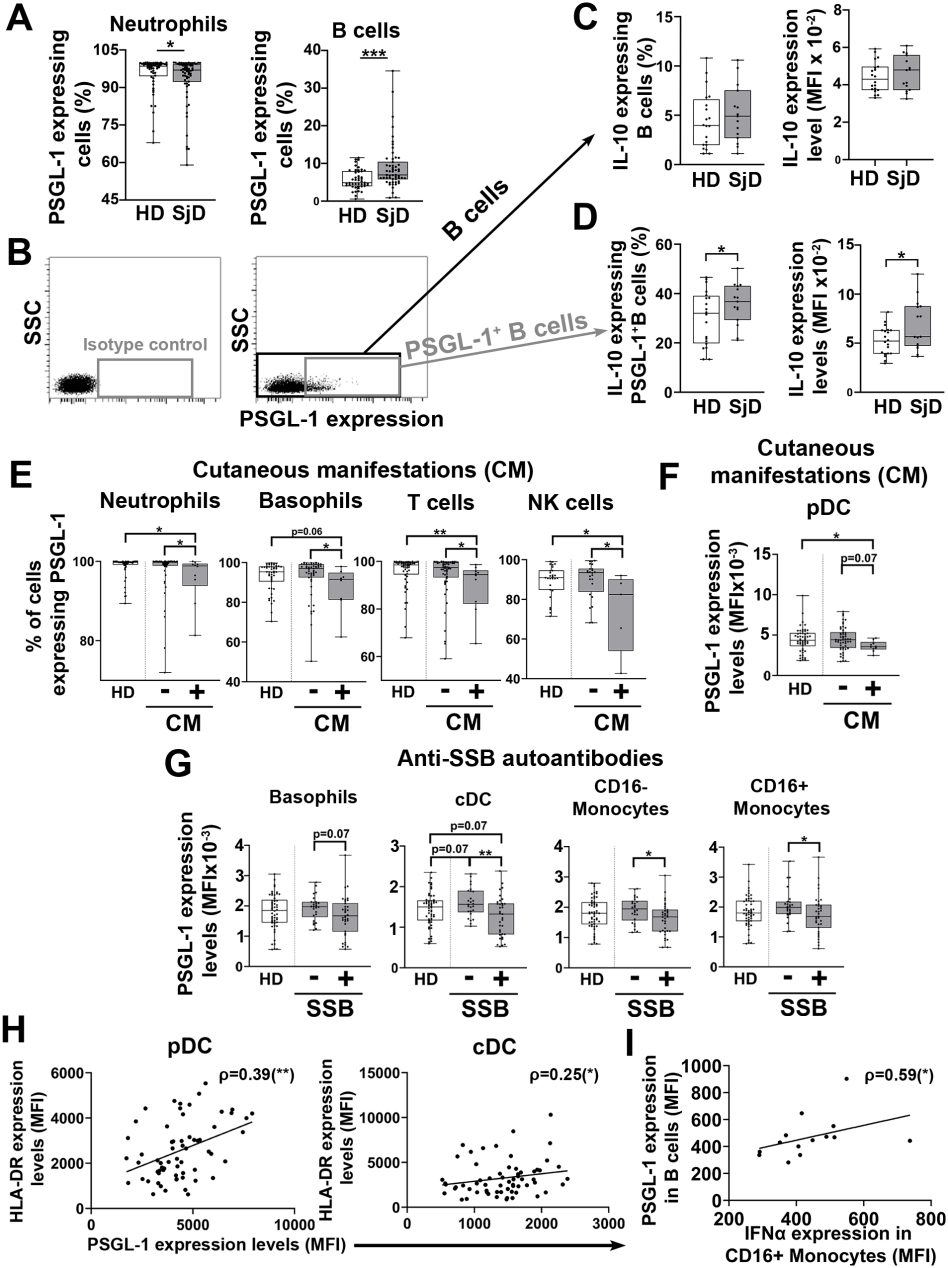
PSGL-1 expression in circulating leukocytes of SjD patients. **(A)** Percentage of PSGL-1 expressing neutrophils (left pannel) and B cells (right pannel) in SjD patients compared to HD. **(B)** Representative flow cytometry dot plots showing B cells: Isotype control (left panel) and PSGL-1^+^ B cells (right panel). **(C)** IL-10 expression in total B cell population: Percentage of IL-10-expressing B cells (left panel) and IL-10 MFI (right panel). **(D)** IL-10 expression in PSGL-1^+^-B cells: percentage of IL-10-expressing cells (left panel) and IL-10 MFI (right panel). **(E, F)** Expression of PSGL-1 in different immune cell populations in HD and SjD without (–) or with (+) cutaneous manifestations: **(E)** Percentage of PSGL-1 expressing neutrophils, basophils, T cells and NK cells. **(F)** PSGL-1 expression levels (MFI) in pDC. **(G)** PSGL-1 expression levels (MFI) in basophils, cDC, CD16^-^ and CD16^+^ monocytes in HD and SjD without (–) or with (+) anti-SSB autoantibodies. **(H)** Correlation between HLA-DR and PSGL-1 expression levels in pDC (left panel) and in cDC (right panel) of SjD patients. I) Correlation between PSGL-1 expression levels in B cells with IFNα expression levels in CD16^+^ Monocytes. Bivariate correlations were assessed using Spearman’s rank correlation coefficient. *p<0.05, **p<0.01, ***p<0.001. cDC, Conventional dendritic cells; CD16^-^ Monocytes, Classical monocytes; CD16^+^ Monocytes, Non classical monocytes; CM, Cutaneous Manifestations; HD, Healthy donors; pDC, Plasmacytoid dendritic cells; SSB, Anti-SSB autoantibodies; SjD, Sjögren’s disease. Sample sizes: **(A)** Neutrophils: HD n=54, SjD n=63; B cells: HD n=42, SjD n=51. **(C, D)** HD n=20, SjD n=15. **(E, F)** Neutrophils, basophils and pDC: HD n=54, CM (–) n=53, CM (+) n=10. T cells: HD n=47, CM (–) n=49, CM (+) n=8. NK cells: HD n=27, CM (–) n=24, CM (+) n=5. **(G)** HD n=54, SSB (–) n=26, SSB (+) n=35. **(H)** pDC-cDC: n=63. **(I)** n=14.

We next analyzed PSGL-1 expression across circulating immune cell populations in SjD patients. We found that SjD patients had reduced percentage of PSGL-1^+^ neutrophils and increased percentage of PSGL-1^+^ B cells (**Figure 4A**). Nevertheless, serum PSGL-1 levels did not differ between SjD patients and HD (p = 0.27) (**Supplementary Figure 4B**). Likewise, stratification according to ESSDAI (<5 vs ≥5) did not reveal statistically significant differences.

Previous studies have identified PSGL-1^+^IL-10^+^ B cells as a subset of B cells, that modulate the production of IgG and the activation of B cells, in *in vitro* experiments with human B cells (18, 27, 28). Considering the immunoregulatory role of PSGL-1 and IL-10^+^, we evaluated IL-10 expression in B cells of SjD patients (**Figure 4B**). While IL-10 expression in total B cells was similar between HD and patients (**Figure 4C**), the PSGL-1^+^ B cell population from SjD patients exhibited higher IL-10 expression (both percentage and MFI) compared to those from HD (**Figure 4D**).

Importantly, we found that lower percentages of circulating PSGL-1 expressing neutrophils, basophils, T cells and NK cells, as well as lower PSGL-1 expression level in pDC, were associated with the presence of cutaneous manifestations (**Figures 4E, F**), and no differences with HD were found in patients without cutaneous manifestations. Additionally, lower expression level of PSGL-1 in basophils, cDC and monocytes (both CD16^+^ and CD16^-^), were associated with the presence of anti-SSB autoantibodies (**Figure 4G**). In addition, patients without anti-SSB autoantibodies showed a trend toward higher expression levels of PSGL-1 in cDC (p=0.07).

Furthermore, we have performed correlation analyses of PSGL-1 expression levels and found a positive correlation with HLA-DR expression levels in pDC (ρ=0.39) and cDC (ρ=0.25) (**Figure 4H**). We also identified a positive correlation (ρ=0.59) between PSGL-1 expression levels in B cells and IFNα expression levels in CD16^+^ monocytes (**Figure 4I**).

### 3.5 Potential diagnostic value for SjD of the ratio between the percentage of circulating PSGL-1-expressing B cells and the percentage of circulating pDC

**Figure 5:**
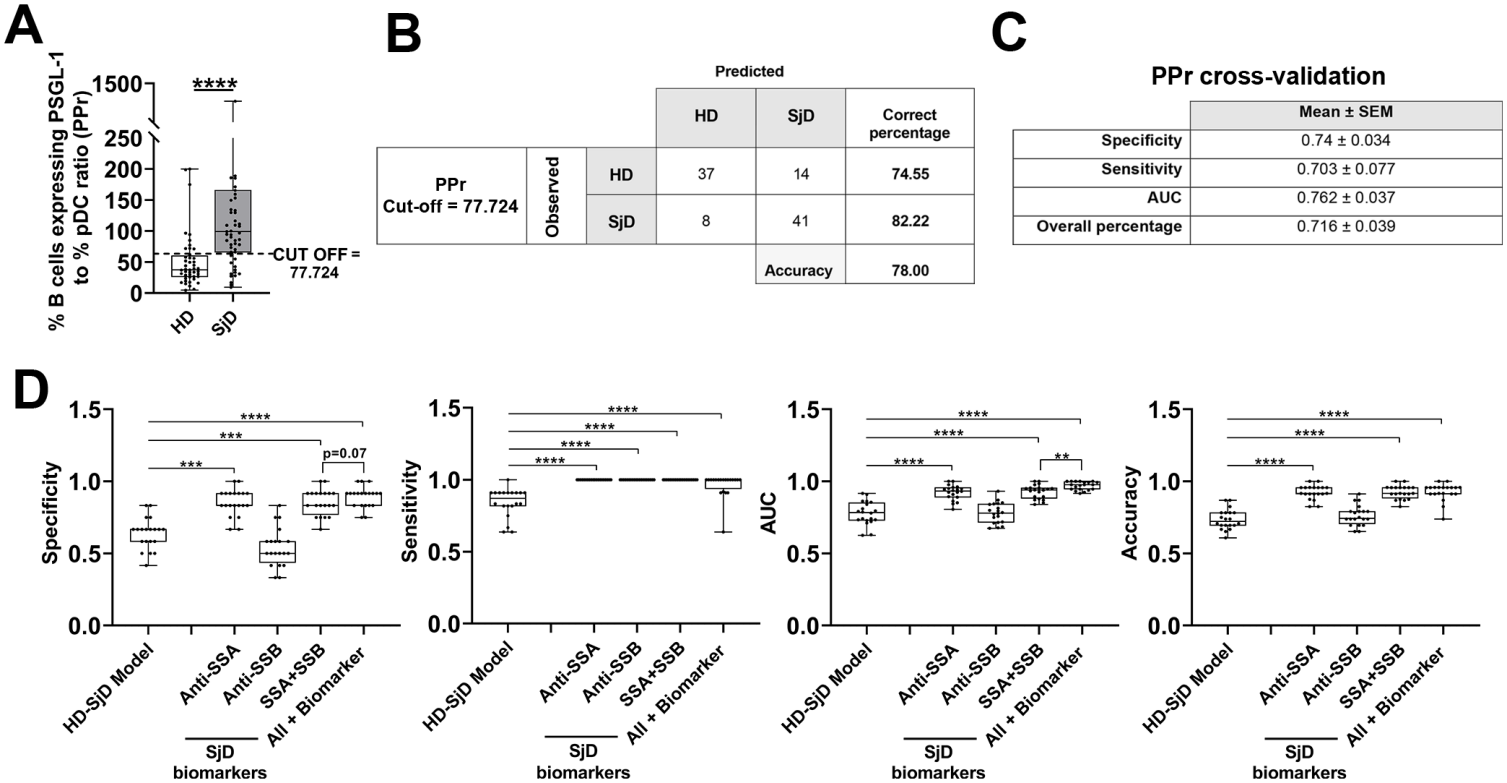
Diagnostic potential of PPr biomarker to discriminate between HD and SjD. **(A)** Boxplot of the PPr in HD and SjD patients, with optimal cut-off determined using Youden’s index (HD n=48, SjD n=55). **(B)** Confusion matrix summarizing the diagnostic performance of the PPr for distinguishing HD and SjD patients, showing sensitivity, specificity, and accuracy from BLR. **(C)** Fivefold cross-validation of the logistic regression model showing mean ± SEM for sensitivity, specificity, AUC, and accuracy. **(D)** Comparative diagnostic performance of the PPr versus classical serological markers for SjD (anti-SSA, anti-SSB), including models combining these biomarkers with or without the PPr. Boxplots show distribution of specificity, sensitivity, AUC, and accuracy across 20 cross-validation iterations. **p<0.01, ***p<0.001, ****p<0.0001. AUC, Area under the curve; HD, Healthy donors; pDC, Plasmacytoid dendritic cells; SjD, Sjögren’s disease.

We found that, compared to HD, SjD patients exhibited a marked decrease in circulating pDC (**Figure 1G**) and increased percentage of PSGL-1-expressing-B cells (hereafter, PS1B) (**Figure 4A**). We therefore evaluated whether the ratio of PS1B to the percentage of circulating pDC (ratio hereafter referred to as PPr) (**Figure 5A**), could serve as a diagnostic marker for SjD. We performed a multivariable BLR analysis using PPr and adjusting for age and sex as potential confounders. This analysis demonstrated promising discriminatory power between SjD patients and HD, with an optimal cut-off of 77.7, determined by YI (**Figure 5A**), achieving a specificity of 74.6%, sensitivity of 82.2%, and an overall accuracy of 78.0% (**Figure 5B**). Internal cross-validation confirmed stable performance across folds (**Figure 5C**). Importantly, when compared to serological biomarkers for SjD (anti-SSA and anti-SSB autoantibodies), the PPr showed comparable performance and provided incremental value over serology alone, improving AUC and with a trend toward improved specificity (p=0.07) (**Figure 5D**).

### 3.6 Diagnostic potential of PPr to discriminate SjD patients from SSc patients

**Figure 6:**
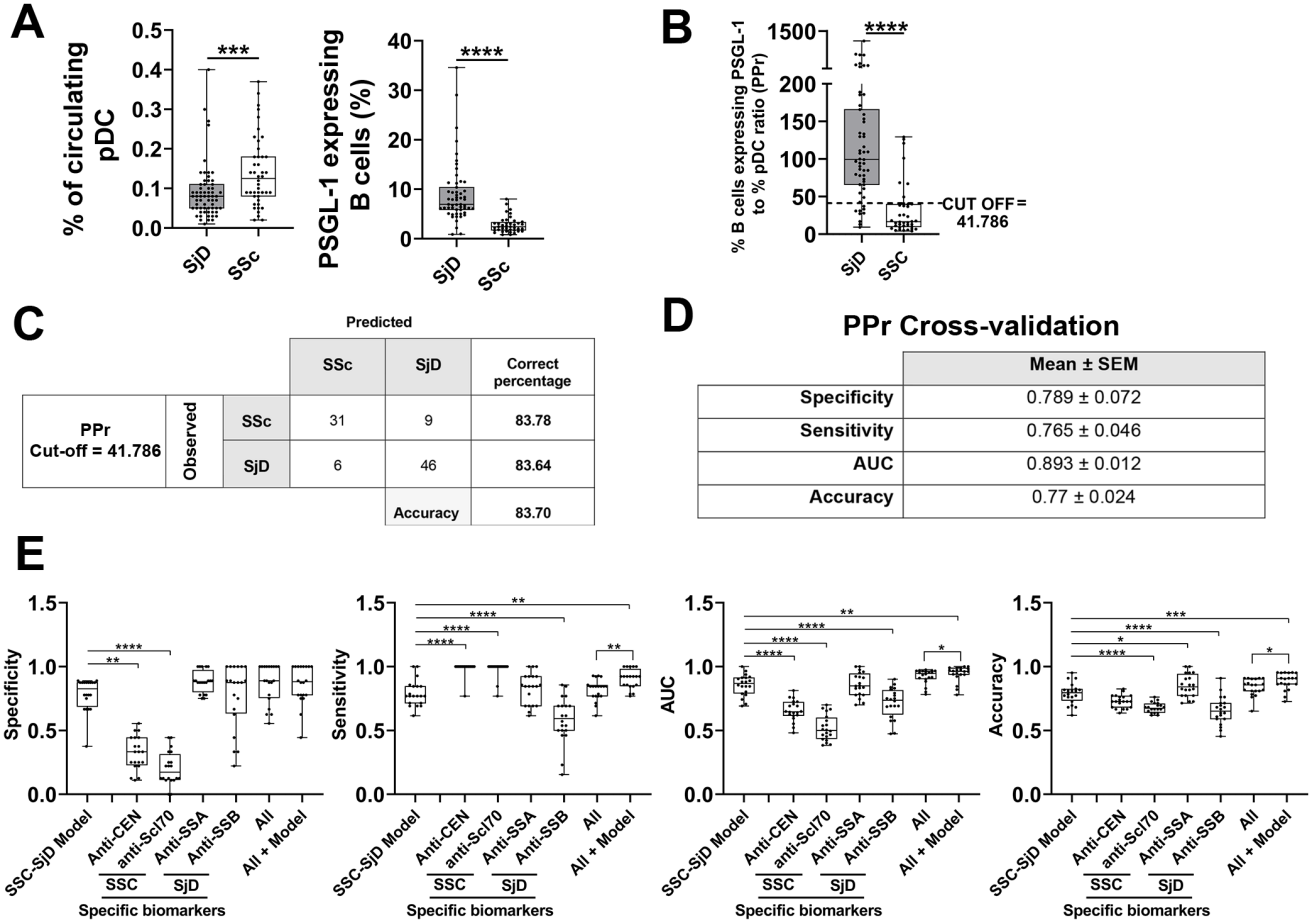
Diagnostic potential of PPr biomarker to discriminate between SjD and SSc. **(A)** Boxplots showing the percentage of circulating pDC (SjD n=63, SSc n=44) and PSGL-1^+^ B (SjD n=55, SSc n=40). **(B)** Boxplot of the PPr in SjD and SSc patients, with optimal cut-off determined using Youden’s index (SjD n=55, SSc n=40). **(C)** Confusion matrix summarizing the diagnostic performance of the PPr for distinguishing SjD and SSc patients, showing sensitivity, specificity, and accuracy from BLR. **(D)** Fivefold cross-validation of the logistic regression model showing mean ± SEM for sensitivity, specificity, AUC, and accuracy. **(E)** Comparative diagnostic performance of the PPr model versus classical serological markers (anti-Scl70, anti-CENP for SSc; anti-SSA, anti-SSB for SjD), including models combining these biomarkers with or without the PPr. Boxplots show distribution of specificity, sensitivity, AUC, and accuracy across 20 cross-validation iterations. *p<0.05, **p<0.01, ***p<0.001, ****p<0.0001. AUC, Area under the curve; pDC, Plasmacytoid dendritic cells; SjD, Sjögren’s disease; SSc, Systemic sclerosis.

SjD and SSc share overlapping features, which complicates early diagnosis. Compared to SjD patients, SSc patients have a higher percentage of circulating pDC and lower PS1B (**Figure 6A**). We therefore evaluated whether the PPr could discriminate between these conditions. We developed a multivariable BLR analysis using PPr, adjusted for age and sex as potential confounders (**Figure 6B**). The optimal diagnostic cut-off, determined by YI, was 41.8 (**Figure 6B**). This model demonstrated strong discriminatory capacity, achieving 83.8% of specificity, 83.6% sensitivity and an overall accuracy of 83.7% (**Figure 6C**). Internal cross-validation confirmed the robustness of the model, showing consistent model performance across sensitivity, specificity, AUC, and accuracy (**Figure 6D**). Compared to established biomarkers, anti-Scl70 and anti-centromere (CEN) for SSc, and anti-SSA/SSB for SjD, PPr showed specificity similar to SjD biomarkers and higher than SSc biomarkers; sensitivity was lower than SSc biomarkers but similar to anti-SSA and higher than anti-SSB. Importantly, combining PPr with all serological biomarkers significantly improved classification metrics, improving the sensitivity, AUC, and accuracy for detecting both diseases (**Figure 6E**).

### 3.7 Diagnostic potential of PS1B to discriminate between SjD patients and SLE patients

**Figure 7:**
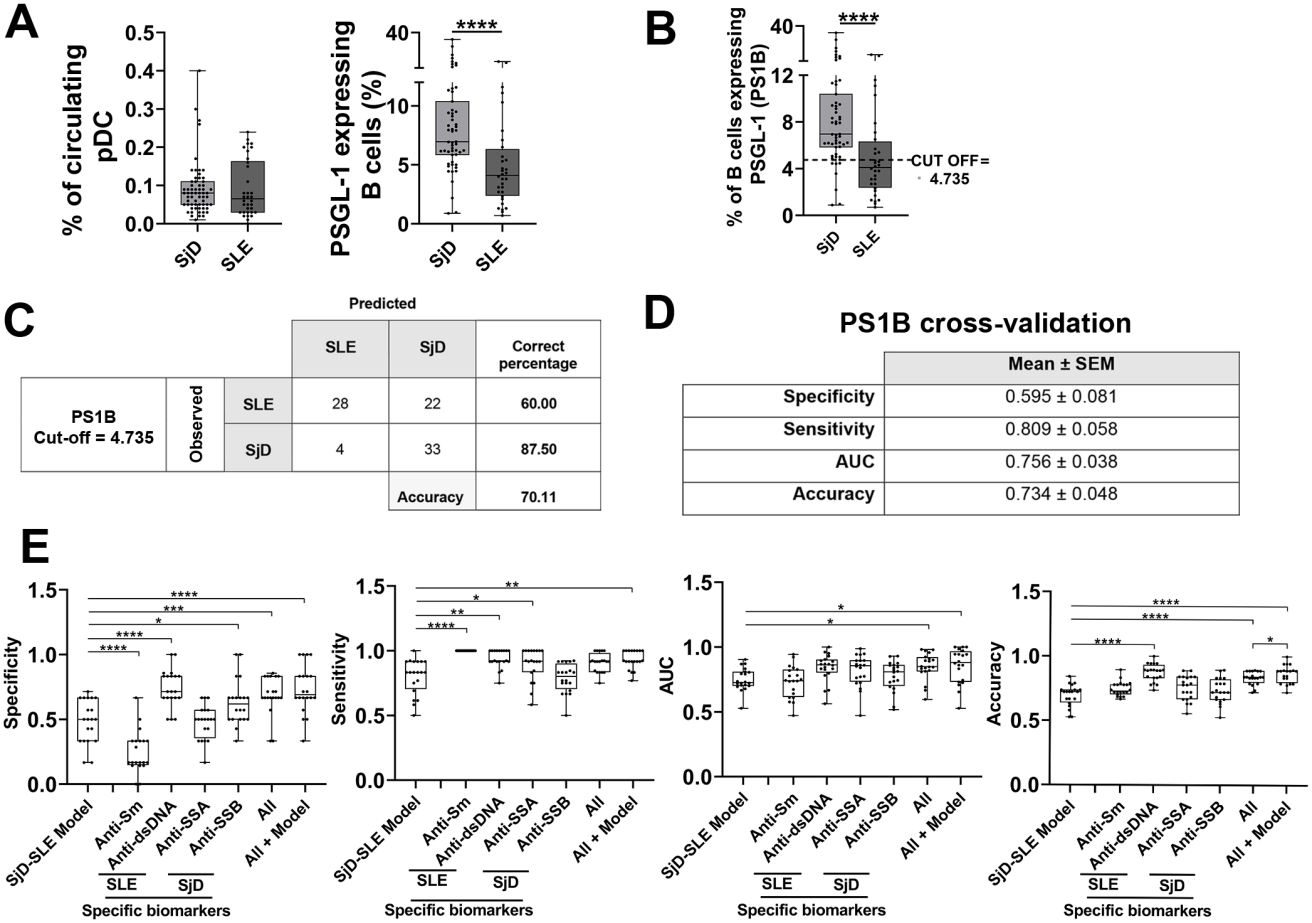
Diagnostic potential of PS1B biomarker to discriminate between SjD and SLE. **(A)** Boxplots showing the percentage of pDC (SjD n=63, SLE n=34) and % of PSGL-1^+^ B cells (SjD n=55, SLE n=32). **(B)** Confusion matrix summarizing the diagnostic performance of the PS1B for distinguishing SjD and SLE patients, showing sensitivity, specificity, and accuracy from BLR. **(C)** Boxplot of the PS1B in SjD and SSc patients, with optimal cut-off determined using Youden’s index. **(D)** Fivefold cross-validation of the logistic regression model showing mean ± SEM for sensitivity, specificity, AUC, and accuracy. **(E)** Comparative diagnostic performance of the PS1B model versus classical serological (anti-Sm and anti-dsDNA for SLE; anti-SSA and anti-SSB for SjD), including models combining these biomarkers with or without the PS1B. Boxplots show distribution of specificity, sensitivity, AUC, and accuracy across 20 cross-validation iterations. *p<0.05, **p<0.01, ***p<0.001, ****p<0.0001. AUC, Area under the curve; pDC, Plasmacytoid dendritic cells; SLE, Systemic lupus erythematosus; SjD, Sjögren’s disease.

Finally, given the considerable clinical and immunological overlap between SjD and SLE, especially in early phases or seronegative cases, we investigated whether our data could help distinguish between these two diseases. The percentages of circulating pDC were similar in SLE and SjD patients. In contrast, PS1B were lower in SLE than in SjD patients (**Figure 7A**), suggesting potential as a discriminatory biomarker. A multivariable BLR analysis, adjusting for age and sex as potential confounders, demonstrated strong discriminatory power for PS1B. An optimal cut-off of 4.7, determined by YI yielded a specificity of 60%, sensitivity of 87.5% and accuracy of 70.1% (**Figures 7B, C**). Five-fold cross-validation confirmed robust model performance across folds (**Figure 7D**). Compared to autoantibody-based biomarkers for SLE (anti-Sm and anti-dsDNA) and SjD (anti-SSA and anti-SSB), inclusion of PS1B in a combined model improved classification accuracy (**Figure 7E**).

### 3.8 DeLong’s test for correlated ROC curve comparisons

We performed a comparison of AUC values for each biomarker across all comparison sets to assess whether the selection of the biomarker was redundant (**Supplementary Figures 5A–C**). Pairwise comparisons of AUCs from correlated ROC curves were conducted using DeLong’s test (**Supplementary Figure 5D**). As shown by this test, PPr is an appropriate biomarker for the HD-SjD and SjD-SSc comparisons. Specifically, for HD-SjD, PPr showed a significant improvement over PS1B (p=0.018) and pDC (p = 0.048). Similarly, for SjD-SSc, PPr showed a significant improvement over PS1B (p=0.019) and pDC (p=3.56 × 10^-5^). In contrast, for the SjD-SLE comparison, DeLong’s test for correlated ROC curves revealed that PS1B has a significantly higher AUC than PPr (p=0.050).

## 4 Discussion

In this study, we have found that SjD patients have reduced numbers of circulating leukocytes. We also observed generalized immune system activation and altered expression of PSGL-1, ADAM8 and IFNα. Notably, low expression levels of ADAM8 in B cells, plasma cells and plasmablasts was associated with disease activity, whereas low expression level of PSGL-1 in pDC and low percentage of PSGL-1-expressing neutrophils, basophils, T cells and NK cells was associated with presence of cutaneous manifestations. Additionally, low expression levels of PSGL-1 in basophils, cDC and monocytes was associated with presence of anti-SSB autoantibodies. Importantly, the PSGL-1^+^ B cell population was increased and exhibited higher IL-10 expression compared to HD, indicating the expansion of a PSGL-1^+^ IL-10-enriched B-cell subset. Based on these alterations, we identified promising cellular biomarkers capable of distinguishing SjD patients not only from HD but also from patients with SLE or SSc.

SjD patients have been reported to exhibit various cytopenias, including mild leukopenia, lymphopenia, and neutropenia (1). We conducted a comprehensive analysis to identify and quantify circulating immune cell populations, revealing a global reduction in circulating leukocytes in SjD patients. Except for plasmablasts and plasma cells, the cell counts of the remaining leukocyte populations were reduced. Interestingly, although cytopenia in SjD patients is commonly attributed either to disease activity or to immunosuppressive therapy, in our cohort we found no association between cytopenia and the ESSDAI score or the use of immunomodulatory therapy. In fact, patients with low disease activity (ESSDAI<5) had lower circulating leukocyte counts than HD, and we found no statistically significant differences between patients with low and moderate-high ESSDAI scores (ESSDAI≥5). Regarding treatment with immunomodulators, the total leukocyte reduction is not affected by their intake and, in the case of MTX or GC, instead of reducing, they raised the number of circulating leukocytes, as described for glucocorticoids which increase the number of neutrophils while reducing the number of lymphocytes, monocytes and eosinophils (29). Taken together, our data indicate that cytopenia is an intrinsic feature of SjD and not secondary to disease activity or a therapy-related effect. Nevertheless, we cannot rule out the possibility that the reduction of some of these circulating populations could be due to their higher infiltration into gland tissue. In this line, published studies have described migration of dendritic cells into inflamed salivary glands (30). Additionally, increased HLA-DR expression has been reported in T cells of SjD patients (31). In our cohort of SjD patients, we observed increased HLA-DR expression levels in CD16^-^ and CD16^+^ monocytes, pDC and B cells and increased percentage of HLA-DR^+^ cells among neutrophils, T cells, NK cells, and basophils. Although rare or present at low levels under homeostatic conditions, increased HLA-DR expression in these non-professional antigen-presenting populations has been described in inflammatory contexts (32–34). These findings underscore the presence of a sustained and chronic inflammatory milieu in Sjögren’s disease.

Regarding IFNα, previous studies have reported increased serum levels of IFNα in SjD patients, with pDC being pointed as the main producers (35, 36) as well as increased transcription of ISGs in the blood of SjD patients (37). In this context, it has also been described that a subtype of cDC2 is increased in SjD patients, has elevated ISG expression and might contribute to SjD pathology through NK activation of a subset of NK cells (38). Additionally, it has been assumed that pDC are the main producers of IFNα in these patients, as occurs in viral infections. However, in autoimmune diseases, the protein level of IFNα produced by different immune cell populations has not been thoroughly assessed. This is an important issue because, in addition to IFNα, ISGs can also be induced by other pathways, including other interferons or cellular stress responses (39–41). Therefore, increased ISG expression in blood cells of SjD patients does not mean that pDC or other DC populations are responsible of increased IFNα production. In fact, our work has found that SjD patients have reduced circulating pDC and cDC, consistent with earlier observations (42). Moreover, our results show that cDC and pDC exhibit increased HLA-DR and reduced IFNα expression levels. As recently proposed, these apparently contradictory findings may reflect a functional shift of pDC toward enhanced antigen-presenting activity at the expense of their capacity to produce type I IFNs (43). This functional change of pDC, together with the reduction of IFNα expression levels in different immune cell populations (monocytes, basophils, pDC, cDC and B cells), may impair antiviral defenses, potentially contributing to increased susceptibility to infections observed in these patients (44, 45), and could help explain the clinical benefit observed in phase III trials using low-dose IFNα therapy (46). Importantly, we found that T cells and CD16^+^ monocytes from SjD patients exhibited higher IFNα expression levels compared to those from HD, and we also observed a positive correlation between PSGL-1 expression levels in B cells and IFNα expression levels in CD16^+^ monocytes. This is consistent with previous studies showing that B-cell survival is compromised by a population of inflammatory monocytes recruited in a type I IFN-dependent manner, thereby weakening antiviral humoral responses and potentially increasing susceptibility to certain viral infections (47). In addition, these findings may be biologically relevant, as type I IFNs can direct B cells toward non-Ig-secreting plasmablasts, which then differentiate into antibody-secreting plasma cells in the presence of IL-6 (26). In line with this, we observed a positive correlation between IFNα expression in T cells and the percentage of circulating plasma cells, which may explain the increased percentage and cell count of plasma cells in our SjD cohort. Interestingly, although it has been described that HCQ modulates the expression of ISGs (48), the production of IFNα by CD16^+^ monocytes from patients treated with HCQ is higher than those from HD and, in the case of T cells, although HCQ-treated group shows a trend toward increased IFNα expression, the statistical significance is lost (p=0.10). However, the differences between patients receiving HCQ and those not receiving HCQ were not statistically significant. Recent clinical trials are exploring antibodies targeting type I IFNs as a potential therapeutic approach. Anifrolumab, a monoclonal antibody directed against the type I IFN receptor, inhibits signaling of all type I IFNs (49). The TULIP-2 clinical trial evaluated anifrolumab in patients with SLE, meeting its primary endpoint and demonstrating beneficial effects in patients with moderate to severe disease (50). This therapy is currently being tested in SjD patients in the ANISE II trial (ClinicalTrials.gov ID NCT05383677). Nevertheless, our data suggest that in SjD anti-type I IFN therapy could be optimized by targeting T cells and CD16^+^ monocytes.

With respect to PSGL-1 and ADAM8, they are two molecules with growing evidence regarding their involvement in various pathologies. Most research on ADAM8 has focused on its role in pulmonary diseases, such as asthma or chronic obstructive pulmonary disease (COPD), and in cancer (51). PSGL-1, in contrast, has been more extensively studied in pathological contexts. Ongoing clinical trials are investigating PSGL-1-targeted therapies for sickle cell vaso-occlusive crisis, asthma and COPD, retinal vasculopathy with cerebral leukoencephalopathy, glioblastoma, and melanoma brain metastases (52). Despite these advances, little is known about the role of these molecules in SjD. We have studied the expression of ADAM8 and PSGL-1 in our SjD cohort and we have found that CD16^+^ monocytes and B cells exhibited decreased ADAM8 expression levels. Moreover, we observed that patients with moderate-high ESSDAI scores had reduced ADAM8 expression levels in B cells, plasma cells, and plasmablasts compared to those with low ESSDAI scores. Notably, in our study, patients treated with MTX do not show statistically significant differences with HD, suggesting a potential role for ADAM8 dysregulation in SjD pathogenesis and further highlighting dysregulation of the B cell compartment in this disease. Nevertheless, the MTX-treated group should be expanded to achieve stronger results.

In the case of PSGL-1, it is a key adhesion molecule involved in leukocyte trafficking and functions as an immune checkpoint (53). In our SjD cohort, we observed that several circulating cell populations exhibited lower PSGL-1 expression in SjD patients with cutaneous involvement or with anti-SSB autoantibodies, pointing these populations as therapeutic targets. These results are consistent with our previous observations, in which we have identified that patients with active SLE also had reduced PSGL-1 expression in neutrophils (16), suggesting that this alteration could represent a potential therapeutic target not only in SjD but also in SLE. Consistently, previous studies have shown that PSGL-1 deficiency can exacerbate tissue damage, such as accelerating bleomycin-induced lung fibrosis and inflammation in mice (54) or aggravating dextran sulphate sodium (DSS)-induced colon inflammation (55). These observations underscore the role of PSGL-1 as a tolerance molecule, whose deficiency may promote uncontrolled activation of immune cells and autoimmunity. Remarkably, we have found that in pDC and cDC, the expression of PSGL-1 and HLA-DR shows a positive correlation, coinciding with previous work describing that activation of monocyte-derived DC increases both, HLA-DR and PSGL-1 expression (13). In contrast, the percentage of B cells expressing PSGL-1 was markedly increased in our cohort of SjD patients. This population exhibited elevated IL-10 production, suggesting the expansion of this PSGL-1^+^ IL-10 enriched B cell subset. Nevertheless, although IL-10 is classically anti-inflammatory, chronic or dysregulated IL-10 signaling can paradoxically promote B-cell activation, plasmablast and plasma cell differentiation, and autoantibody production (56, 57). Additionally, IL-10 produced by B cells has been reported to support malignant B-cell survival and shape the tumor microenvironment in diffuse large B-cell lymphoma, influencing the effectiveness of immunotherapy (58). PSGL-1 itself is highly expressed in several hematologic malignancies, including T-cell acute lymphoblastic leukemia, acute myeloid leukemia, and multiple myeloma, with higher expression correlating with worse outcomes (59). In our cohort, plasma cells were increased, a population whose expansion has been linked to disease progression in SjD (60) and is enriched in certain B-cell non-Hodgkin lymphomas, which are more frequent in SjD patients (61). Taken together, these findings suggest that the expansion of the PSGL-1^+^ IL-10-enriched B-cell subset in SjD might contribute to autoimmunity and plasma cell expansion. This dysregulated population of PSGL-1-expressing B cells might have a potential contribution to an immune environment associated with increased lymphoma risk in Sjögren’s disease.

Treatment of SjD remains challenging due to heterogeneous clinical manifestations. Even with advances in understanding the disease, management has changed little in recent decades (62). Although B cells are increasingly recognized as central to SjD pathogenesis, biologics such as rituximab (RTX) have not achieved the expected outcomes (62). More recently, ianalumab, a B-cell-targeted biologic therapy currently under evaluation, offers a more selective approach than RTX (63). Ianalumab blocks BAFF receptors, thereby inhibiting B-cell survival and activation signals, but also induces B-cell depletion via antibody-dependent cellular cytotoxicity. These findings highlight that current B-cell-depleting therapies are largely non-selective, whereas more precise strategies, such as ianalumab may offer improved therapeutic benefit in SjD. In line with this, we hypothesize that PSGL-1^+^ B cells could serve as a novel therapeutic cellular target. Therefore, strategies that selectively modulate the presence, function or trafficking of PSGL-1^+^ B-cells could provide a more precise approach, preserving beneficial regulatory activity while limiting pathogenic or oncogenic potential. In this regard, we also explored the importance of PSGL-1^+^ B cells (PS1B) in SjD, as they may be relevant not only as a potential therapeutic target but also for diagnosis; BLR identified percentage of circulating pDC and the PS1B as the most relevant variables capable of distinguishing SjD from HD, although neither parameter alone was sufficiently robust. Therefore, we explored whether their ratio (PPr) could improve discriminatory power. Indeed, PPr successfully distinguished SjD from HD with 78.0% accuracy. The PPr complemented available serological markers, improving their ability to discriminate SjD patients from HD. Furthermore, since SLE and SSc share some clinical features with SjD, delaying diagnosis of these conditions until the disease is fully established and organ damage may already be present (2), we studied whether PPr and PS1B could help to discriminate SjD from these other autoimmune conditions. Importantly, the PPr was particularly effective in distinguishing SjD from SSc, reaching 83.7% accuracy, comparable to currently available disease-specific serological markers (anti-CEN and anti-Scl70). In addition, we found that whereas the percentage of circulating pDC was similar in SjD and SLE, the PS1B showed strong classification capacity (70.1% accuracy) to discriminate both diseases, contributing to diagnostic power when integrated with conventional SLE markers (anti-dsDNA and anti-Sm) and SjD biomarkers (anti-SSA and anti-SSB). The relevance of these findings lies in the diagnostic gap that persists in seronegative patients, where antibody-based biomarkers may be absent or inconclusive (64, 65). Hence, the integration of these new cellular biomarkers with the established serological biomarkers represents a complementary approach that could facilitate earlier and more accurate diagnosis.

The main limitations of this study are the small sample size and the focus on patients with established disease, which restricts conclusions about the potential of these cellular biomarkers for early diagnosis. This observational study included 65 SjD, 34 SLE, 44 SSc and 56 HD, with smaller subsets analyzed for some immune populations.

In summary, this study identifies dysregulated expression of ADAM8 and PSGL-1 in circulating cells of SjD patients, which is associated with important clinical manifestations, thereby supporting their potential relevance as therapeutic targets for this disease. Of note, we observed increased percentage of PSGL-1^+^ B cells, and using this population, we defined two novel cellular biomarkers, PS1B and PPr, which could serve as diagnostic indicators not only for SjD but also to distinguish SjD from SSc or SLE. This integrative approach, combining cellular and serological markers, supports earlier and more accurate disease identification and emphasizes the clinical relevance of PSGL-1^+^ B cells as both diagnostic and potential therapeutic target.

## Data Availability

The raw data supporting the conclusions of this article will be made available by the authors, without undue reservation.
