# Supplementary material for: Dysregulated expression of PSGL-1 and ADAM8 in Sjögren’s disease: potential contribution to disease pathogenesis
Source: Front Immunol. 2026 Jul 22;17:1848689. doi: 10.3389/fimmu.2026.1848689 (PMC13445602; doi:10.3389/fimmu.2026.1848689)
Supplement: Supplementary file 1 [file DataSheet1.docx]

**SUPPLEMENTARY MATERIAL**

# 1 Supplementary Figures

**Supplementary Figure 1. Histograms showing isotype controls, FMO control and expression of PSGL-1, HLA-DR, ADAM8, IFNα and IL-10.** **A)** Isotype controls corresponding to FITC, PE, V450 channels. **B)** Expression profile of ADAM8, PSGL-1 and HLA-DR on leukocytes analyzed by flow cytometry. Each upper graph shows the corresponding isotype control **C)** Strategy to determine IFNα positivity: Intracellular PE isotype control, FMO control and positive histograms. **D)** Strategy to determine IL-10 positivity: Intracellular V450 Isotype control and positive IL-10 staining. **FMO:** Fluorescence-Minus-One.

**Supplementary Figure 2. Gating strategies. A)** Gating strategy for myeloid populations. Neutrophils were identified as CD16^+^. Monocytes were identified as CD14^+^, and classical (CD16^-^ CD14^+^, CD16^-^ Monocytes) and non-classical monocytes as (CD16^+^ CD14^+^, CD16^+^ Monocytes) were analyzed. Basophils were identified in the low SSC CD14^-^ gate, as HLA-DR⁻CD123⁺. pDC and cDC were identified after excluding basophils: pDC as HLA-DR^+^, CD11c^-^, CD123^high^ and cDC as HLA-DR^+^, CD11c^+^, CD123^low^. **B)** Gating strategy for lymphoid populations. Within the lymphoid gate (low SSC and FSC), B cells and T cells were defined as CD19⁺ and CD3⁺, respectively. NK cells were identified as CD56⁺ within the CD19⁻CD3⁻ double-negative fraction. Plasmablasts and plasma cells were defined within the CD19⁺ gate as CD19⁺CD38^+^ and CD19^low^CD38⁺, respectively. **cDC:** Conventional dendritic cells; **CD16^+^ Monocytes:** Non-classical monocytes; **CD16^-^ Monocytes:** Classical Monocytes; **NK:** Natural killer; **pDC:** Plasmacytoid dendritic cells.

**Supplementary Figure 3. Hematological parameters and IFNα and ADAM8 expression levels in circulating immune cell populations of SjD patients and HD.** **A-D)** Hematological parameters measured by an automated hematology analyzer in HD and SjD patients: **(A)** HCT, **(B)** HGB, **(C)** RBC, and **(D)** PLT. **E)** MFI of IFNα expression in neutrophils and NK cells. **F)** MFI of ADAM8 expression in neutrophils, CD16^-^ monocytes, basophils, cDC, pDC, T cells and NK cells. **cDC:** Conventional dendritic cells**; HD:** Healthy donors**; HCT:** Hematocrit**; HGB:** Hemoglobin**; pDC:** Plasmacytoid dendritic cells**; PLT:** Platelets**; RBC:** Red blood cells**; SjD:** Sjögren’s disease. **Sample sizes: A-D)** HD n=32, SjD n=52; **E)** Neutrophils: HD n=47, SjD n=57; NK cells: HD n=27, SjD n=30. **F)** Neutrophils, CD16^-^ monocytes, basophils, cDC, pDC and T cells: HD n=51, SjD n=60; NK cells: HD n=27, SjD n=29. *p<0.05.

**Supplementary Figure 4. Serum levels of ADAM8 and PSGL-1. A)** Serum levels of ADAM8 (pg/mL) in patients with SjD and HD, including stratification according to ESSDAI (<5 and ≥5). **B)** Serum levels of PSGL-1 (ng/mL) in patients with SjD and HD, including stratification according to ESSDAI (<5 and ≥5). **HD:** healthy donors; **SjD**: Sjögren’s disease; **ESSDAI:** EULAR Sjögren’s Syndrome Disease Activity Index. **Sample sizes:** **A)** HD n=15, SjD n=24 (ESSDAI <5 n=21; ESSDAI ≥5 n=3). **B)** HD n=15, SjD n=25 (ESSDAI <5 n=21; ESSDAI ≥5 n=4).

**Supplementary Figure 5. ROC curves and DeLong’s tests. A-C)** ROC curves for PPr and its individual components (pDC and PS1B). The right panel shows the overlaid ROC curves, including the AUC values of each biomarker. **A)** ROC curves of biomarkers discriminating HD from SjD patients. **B)** ROC curves of biomarkers discriminating SjD from SLE patients. **C)** ROC curves of biomarkers discriminating SjD from SSc patients. **D)** DeLong’s test for pairwise comparisons of correlated ROC curves. **HD:** Healthy donors; **SjD:** Sjögren’s disease. **SSc:** Systemic sclerosis. **SLE:** Systemic lupus erythematosus.

# 2 Supplementary Tables

**Supplementary Table 1.** Clinical features of HD and SjD patients participating in the study.

| **Clinical features** | |
| --- | --- |
| **Healthy donors** | |
| Female/Male; n (%) / n (%) | 48 (86) / 8 (14) |
| Age (years); mean [min-max] | 62.5 [21-90] |
| **SjD patients** | |
| Female/Male; n (%) / n (%) | 60(92) / 5(8) |
| Age (years); mean [min-max] | 61.98 [27-92] |
| **Disease activity indexes** |  |
| Low (ESSDAI < 5); n [min-max] | 54 [0-4] |
| *Articular; n (%)* | 8 (15) |
| *Hematological; n (%)* | 4 (7) |
| *Biological; n (%)* | 21 (39) |
| Moderate – High (ESSDAI ≥ 5); n [min-max] | 10 [5-19] |
| *Lymphoma; n (%)* | 2 (20) |
| *Glandular; n (%)* | 1 (10) |
| *Articular; n (%)* | 4 (40) |
| *Cutaneous; n (%)* | 3 (30) |
| *Pulmonary; n (%)* | 2 (20) |
| *Renal; n (%)* | 1 (10) |
| *Hematological; n (%)* | 2 (20) |
| *Biological; n (%)* | 7 (70) |
| *Patients with ≥ 2 extraglandular domains; n (%)* | 8 (80) |
| CLINESSDAI; mean [min-max] | 1.78 [0-19] |
| ESSPRI; mean [min-max] | 5.38 [0.67-10] |
| **Diagnostic tests; n (%)** |  |
| Minor salivary gland biopsy | 41 (63.08) |
| *Positive; n (%)* | 32 (78.05) |
| *Negative; n (%)* | 2 (4.88) |
| *Indeterminate; n (%)* | 7 (17.07) |
| Schirmer’s test | 20 (30.77) |
| Unstimulated salivary flow rate | 25 (38.46) |
| **Autoantibodies profile n (%)** |  |
| ANA + | 61 (97) |
| Anti-SSA + | 52 (84) |
| Anti-SSB + | 35 (56) |
| RF + | 31 (52) |
| **Extraglandular manifestations; n (%)** |  |
| Articular | 35 (54) |
| Renal | 4 (6) |
| Vascular | 6 (9) |
| Cutaneous | 10 (15) |
| *Raynaud’s phenomenon; n(%)* | 6 (60) |
| *Xerosis; n(%)* | 1 (10) |
| *Purpura; n(%)* | 1 (10) |
| *Ulcerative cellulitis; n(%)* | 1 (10) |
| *Polymorphous light eruption; n(%)* | 1 (10) |
| Pulmonary | 5 (8) |
| Neuronal | 2 (3) |
| Lymphoma | 2 (3) |
| **Other clinical parameters** |  |
| B2MG; mean [min-max] | 2.28 [1.2-4.2] |
| *0.8-2.2 mg/L (normal values); n (%)* | 30 (57) |
| *>2.2 mg/L (high values); n (%)* | 23 (43) |
| **Treatment; n (%)** |  |
| Rituximab | 3 (5) |
| Hydroxychloroquine | 29 (45) |
| Methotrexate | 10 (15) |
| Glucocorticoids | 7 (11) |
| Mycophenolate mofetil | 4 (6) |

**Abbreviations:** **ANA:** Antinuclear antibodies; **Anti-SSA:** Anti Sjögren’s Syndrome Antigen A antibodies; **Anti-SSB:** Anti Sjögren’s Syndrome Antigen B antibodies; **B2MG:** β2-microglobulin; **CLINIESSDAI:** Clinical European Sjögren’s Syndrome Disease Activity Index; **ESSDAI:** EULAR Sjögren’s Syndrome Disease Activity Index; **ESSPRI:** European Sjögren’s Syndrome Patient Reported Index; **HD:** Healthy donors; **RF:** Rheumatoid Factor; **SjD:** Sjögren’s disease.

**Supplementary Table 2.** Clinical features of SLE patients participating in the study.

| **Clinical features** | |
| --- | --- |
| **SLE patients** | |
| Female/Male; n (%) / n (%) | 32 (86) / 5 (14) |
| Age (years); mean [min-max] | 46.9 [19-77] |
| **Disease activity/chronicity indexes** |  |
| Low (SLEDAI < 4); n (%) | 21 (66) |
| Moderate – High (SLEDAI ≥ 4) n (%) | 11 (34) |
| SLICC; mean [min-max] | 0.64 [0-4] |
| **Autoantibodies profile** |  |
| ANA +; n (%) | 31 (97) |
| Anti-dsDNA +; n (%) | 25 (71) |
| Anti-Sm +; n (%) | 7 (24) |
| Anti-SSA +; n (%) | 10 (27) |
| Anti-SSB +; n (%) | 4 (11) |
| **Organ involvement; n (%)** |  |
| Cutaneous | 28 (78) |
| Articular | 30 (81) |
| Pulmonary | 11 (31) |
| Renal | 21 (58) |
| Central nervous system | 16 (44) |
| Cardiac involvement | 9 (25) |
| **Treatment; n (%)** |  |
| Azathioprine | 18 (50) |
| Glucocorticoids | 26 (74) |
| Cyclophosphamide | 4 (11) |
| Hydroxychloroquine | 33 (92) |
| Methotrexate | 9 (25) |
| Mycophenolate mofetil | 13 (36) |
| Rituximab | 9 (25) |

**Abbreviations:** **ANA:** antinuclear antibodies; **Anti-ds-DNA:** Anti-double stranded DNA antibodies; **Anti-Sm:** Anti Smith antibodies; **Anti-SSA:** Anti Sjögren’s Syndrome Antigen A antibodies; **Anti-SSB:** Anti Sjögren’s Syndrome Antigen B antibodies; **SLE:** Systemic lupus erythematosus; **SLEDAI:** SLE disease activity index; **SLICC:** Systemic Lupus International Collaborating Clinics.

**Supplementary Table 3.** Clinical features of SSc patients participating in the study.

| **Clinical features** | |
| --- | --- |
| **SSc patients** | |
| Female/Male; n (%) / n (%) | 42 (95) / 2 (5) |
| Age (years); mean [min-max] | 59.14 [27-82] |
| **Clinical subtypes; n (%)** |  |
| lcSSc | 28 (65) |
| dcSSc | 14 (33) |
| sineSSc | 1 (2) |
| **Autoantibodies profile; n (%)** |  |
| ANA + | 39 (91) |
| Anti-CEN + | 15 (35) |
| Anti-Scl70 + | 10 (23) |
| Anti-SSA + | 7 (18) |
| Anti-SSB + | 2 (5) |
| **Organ involvement; n (%)** |  |
| Esophageal | 25 (63) |
| Musculoskeletal | 20 (50) |
| Pulmonary | 11 (28) |
| Heart | 34 (85) |
| **Treatment; n (%)** |  |
| Cyclophosphamide | 2 (5) |
| Rituximab | 2 (5) |
| Methotrexate | 11 (25) |
| Mycophenolate mofetil | 5 (11) |
| Azathioprine | 2 (5) |
| Hydroxychloroquine | 4 (9) |
| Glucocorticoids | 12 (27) |

**Abbreviations:** **ANA:** Antinuclear antibodies; **Anti-CEN:** Anti-centromere antibodies; **Anti-Scl70:** Anti-topoisomerase I antibodies; **Anti-SSA:** Sjögren’s Syndrome Antigen A antibodies; **Anti-SSB:** Sjögren’s Syndrome Antigen B antibodies; **dcSSc:** Diffuse cutaneous systemic sclerosis; **lcSSc:** Limited cutaneous systemic sclerosis; **sineSSc:** Sine systemic sclerosis; **SSc:** Systemic sclerosis.

**Supplementary Table 4.** Antibodies and probes for flow cytometry.

| **Antibody** | **Fluorochrome** | **Dilution** | **Company** | **Reference** |
| --- | --- | --- | --- | --- |
| Anti CD14 | FITC | 1:100 | BD Pharmingen | 561712 |
| Anti CD3 | FITC | 1:100 | BD Pharmingen | 9189183 |
| Anti CD3 | PE | 1:100 | BD Pharmingen | 345765 |
| Anti CD162 (PSGL-1) | PE | 1:100 | BD Pharmingen | 556055 |
| Anti IFNα | PE | 1:100 | Miltenyi Biotec | 130-116-873 |
| Anti CD11c | PECy7 | 1:100 | BD Pharmingen | 561356 |
| Anti CD56 | PECy7 | 1:100 | BD Pharmingen | 557747 |
| Anti CD38 | APC | 1:200 | Biolegend | 303510 |
| Anti CD14 | APC | 1:100 | Biolegend | 301808 |
| Anti CD123 | APC | 1:100 | Biolegend | 306012 |
| Anti CD19 | APCCy7 | 1:100 | Biolegend | 302218 |
| Anti CD16 | APCCy7 | 1:100 | BD Pharmingen | 560195 |
| Anti CD14 | Pacific Blue | 1:100 | Biolegend | 301828 |
| Anti CD16 | Pacific Blue | 1:100 | BD Pharmingen | 558122 |
| Anti HLA-DR | Pacific Blue | 1:200 | Biolegend | 307624 |
| Anti IL-10 | Brilliant Violet 421 | 1:100 | Biolegend | 501421 |
| Anti human ADAM8 | Pure | 1:100 | R&D Systems | AF1031 |
| Donkey anti-Goat IgG (H+L) Antibody Alexa 488 | FITC | 1:100 | Invitrogen | A11055 |
| **Isotypes** | | | | |
| Isotype FITC | FITC | 1:1200 | Biolegend | 400110 |
| Isotype PE | PE | 1:1200 | BD Pharmingen | 556656 |
| Isotype PECy7 | PECy7 | 1:1200 | Biolegend | 410721 |
| Isotype APC | APC | 1:1200 | BD Pharmingen | 554681 |
| Isotype APC-Cy7 | APC-Cy7 | 1:1200 | Biolegend | 400128 |
| Isotype Pacific Blue | Pacific Blue | 1:24000 | Biolegend | 400235 |
| **Other reagents** | | | | |
| FACS Lysing solution 10x concentrate* |  | 1:10 | BD Pharmingen | 349202 |
| CountBright™ Absolute Counting Beads |  |  | Invitrogen | C36950 |
| Rainbow Calibration Particles, 8 Peaks (3.0–3.4 µm) |  |  | BD Pharmingen | 559123 |

All of them diluted in PBS 1x. *: Diluted in distilled water.
